# Dosage Adjustments for Chemotherapy and Targeted Therapies in Colorectal and Pancreatic Cancer Patients with Hepatic Impairment

**DOI:** 10.7759/cureus.2798

**Published:** 2018-06-13

**Authors:** Sidra Khalid, Aariez Khalid, Bernadette A Clark, Abdo Haddad, Timothy PP Spiro, Hamed Daw

**Affiliations:** 1 Internal Medicine Residency, Fairview Hospital, Cleveland Clinic, Cleveland, USA; 2 Biomedical Science, University of Guelph, Guelph, CAN; 3 Oncology, Fairview Hospital, Cleveland Clinic, Cleveland, USA; 4 Department of Hematology and Oncology, Fairview Hospital, Cleveland Clinic, Cleveland, USA

**Keywords:** hepatic impairment, colon cancer, rectal cancer, pancreatic cancer, chemotherapy, targeted therapy

## Abstract

There are many novel chemotherapeutic options and targeted therapies available for the treatment of colorectal and pancreatic cancer. Patients with these cancers often have hepatic impairment either from the metastasis to the liver or from the chemotherapy or targeted therapies used to treat the disease. It is important to describe the effects of these agents in patients with hepatic impairment. This article will review the dosage recommendations for the chemotherapy regimens and targeted therapies in colorectal and pancreatic cancer patients in the setting of hepatic impairment.

## Introduction and background

Hepatic impairment in colorectal and pancreatic cancer often occurs in the setting of liver metastasis and/or because of the use of chemotherapy and targeted therapies. When clinicians are faced with managing metastatic colorectal and pancreatic cancer in patients with hepatic impairment, there is a need to adjust the doses of chemotherapy and targeted therapy to avoid toxicity. In this article, we will review chemotherapy and targeted therapies used for colorectal and pancreatic cancer along with their mechanisms of action. We will advise as to whether dose adjustments are required and, if necessary, we will provide recommendations based on the degree of hepatic impairment.

## Review

Medications without recommendations for dose adjustments

There are many options available for treating colorectal and pancreatic cancer patients with hepatic impairment. Some of the medications do not require dose adjustments. Many medications are not metabolized in the liver and the pharmacokinetics and pharmacodynamics are not expected to be affected by impaired liver function. Other medications were not studied in patients with hepatic impairment in clinical trials and therefore recommendations for adjustments are not made (Table 1).

**Table 1:** Medications that do not require dose adjustment for hepatic impairment.

Medication	Drug Class	US Food and Drug Administration (US FDA) approved indications
Bevacizumab	-Vascular endothelial growth factor (VEGF) antibody	-Metastatic colorectal cancer, in combination with 5-fluorouracil for first or second line treatment -Metastatic colorectal cancer in combination with fluoropyrimidine-based chemotherapy
Cetuximab	-Epidermal growth factor receptor (EGFR) antagonist	-EGFR expressing metastatic colorectal cancer after failure of irinotecan and oxaliplatin-based chemotherapy -in combination with irinotecan in EGFR expressing metastatic colorectal cancer in patients who are refractory to irinotecan-based chemotherapy
Panitumumab	-EGFR antagonist	-Single-agent treatment for metastatic colorectal cancer with disease progression on or following fluoropyrimidine, oxaliplatin, and irinotecan chemotherapy regimens
Oxaliplatin	-Platinum-based chemotherapeutic agent	-Used in combination with 5-fluorouracil/leucovorin in adjuvant treatment of stage III colon cancer with complete resection of the primary tumor; treatment of advanced colorectal cancer
Trifluridine-tipiracil *do not use in moderate or severe hepatic impairment	-Nucleoside inhibitor and thymidine phosphorylate inhibitor	-Used in patients with metastatic colorectal cancer who have had a prior treatment with fluoropyrimidine, oxaliplatin, irinotecan-based chemotherapy; anti-VEGF therapy; or if RAS wild-type, an anti-EGFR therapy
Ramucirumab *no data available for severe hepatic impairment	-Monoclonal antibody that binds to vascular endothelin growth factor receptor (VEGFR) 2 -Antiangiogenic	-Used in combination with FOLFIRI (irinotecan, folinic acid, 5-fluorouracil) in metastatic colorectal cancer with progression on or after prior therapy with bevacizumab, oxaliplatin, and fluoropyrimidine
Nivolumab *not studied in moderate to severe hepatic impairment *monitor for immune-mediated hepatitis	-Anti-program cell death protein (PD) 1 human monoclonal antibody	-Mismatch repair deficient (dMMR) and microsatellite instability high (MSI-H) metastatic colorectal cancer that has progressed following treatment with fluoropyrimidine, oxaliplatin, and irinotecan
Ziv-Afibercept *no data available for severe hepatic impairment	-Recombinant fusion protein that consists of VEGF-binding portions from the extracellular domains of human VEGFR 1 and 2 fused to Fc portion of human IgG1 immunoglobulin	-Used in combination with 5-fluorouracil, leucovorin, irinotecan, in metastatic colorectal cancer that is resistant to or has progressed following an oxaliplatin chemotherapy regimen

Medications that require dose adjustments due to hepatic impairment

The chemotherapeutic agents and targeted therapies that require dosage adjustment are mostly metabolized in the liver. When hepatic impairment leads to difficulty in metabolizing the medications and their metabolites, there is an increased risk of toxicity. In order to prevent toxicity, these medications require dose adjustments in the setting of hepatic impairment.

5-fluorouracil

5-fluorouracil is a pyrimidine analogue that is Food and Drug Administration (FDA) approved for colorectal and pancreatic cancer [1]. It inhibits thymidylate synthase which leads to the decreased synthesis of thymidine, a nucleotide used for DNA and RNA synthesis. 5-fluorouracil can cause transient hepatotoxicity through direct intrinsic injury of the hepatocytes. It is metabolized by the liver through the microsomal enzyme system. Production of a toxic intermediate causes the liver injury. 5-fluorouracil administration can lead to a mild elevation of serum aminotransferases. At higher doses delivered by continuous infusion, there can be a rapid development of hepatic coma with hyperammonemia. Patients can develop encephalopathy within 72 hours of receiving 5-fluorouracil. In these cases, 5-fluorouracil should be held and an ammonia lowering therapy should be initiated. Although, there is no recommended dose that can be safely administered after developing hepatic coma and hyperammonemia, patients who develop this complication may be able to tolerate 5-fluorouracil at lower doses when the toxic complications resolve [2].

In a phase 1 study by Fleming et al., 64 patients with organ dysfunction were administered 5-fluorouracil and leucovorin as a 24-hour continuous infusion. They were divided into three cohorts: cohort 1 – creatinine >1.5 mg/dL, normal bilirubin; cohort 2 – bilirubin >1.5 mg/dL, normal creatinine; cohort 3 – bilirubin ≥5.0 mg/dL, normal creatinine. After comparing the results for the three cohorts, it was concluded that patients with elevated bilirubin levels could be safely treated with weekly doses of 5-fluorouracil at 2600 mg/m^2^ with leucovorin 500 mg/m^2^ as a continuous 24-hour infusion [3]. It is recommended liver function tests be drawn at baseline. In moderate to severe hepatic impairment, 5-fluorouracil doses need to be reduced. If bilirubin is <2 times the upper limit of normal (ULN) and aspartate transaminase (AST)/alanine transaminase (ALT) 3-5 times ULN reduce the dose to 75%. If bilirubin is 2-4 times ULN or AST/ALT are 5-10 times ULN reduce the dose to 50-75%. If bilirubin is >4 times ULN or AST/ALT are >10 times ULN, 5-fluorouracil should not be administered [4].

Capecitabine

Capecitabine is a prodrug of 5-fluorouracil. It undergoes hydrolysis in the liver and tissues to become fluorouracil. It is approved as a first-line agent for metastatic colorectal cancer and for stage III colorectal cancer patients who have undergone resection of their primary tumor. Its mechanism of causing hepatotoxicity is due to its conversion into fluorouracil and has been described in this review previously [5]. Mild to moderate hepatic impairment from liver metastasis does increase the AUC (area under the curve) of capecitabine and Cmax (maximum serum concentration) by 60%. However, the active drug (fluorouracil) is not affected. Serum aminotransferase elevations above five times the upper limit occur rarely in <1% patients. Bilirubin elevation mostly indirect hyperbilirubinemia can also be seen, and are usually self-limited and mild [5]. Twelves et al. demonstrated that hepatic metastasis had no clinically significant effect on the pharmacokinetic properties of capecitabine or its metabolites. Capecitabine dose adjustment is not required in mild to moderate hepatic impairment [6]. In the study trial, capecitabine was compared to 5-fluorouracil as adjuvant treatment for stage III colon cancer. In the capecitabine alone group, hyperbilirubinemia was reported in 50/995 patients (all grades) and 20/995 patients developed grade 3 or 4 [7]. When grade 3 or 4 elevations in bilirubin occur, it is recommended that capecitabine be discontinued until bilirubin levels decrease to less than three times the ULN, and then reduce the dose as described in the labeling [8].

Regorafenib

Regorafenib is an oral multi-kinase inhibitor. It inhibits VEGFR 2 and 3, Ret, Kit, platelet-derived growth factor receptor (PDGFR) and Raf kinases, which prevents angiogenesis and tumor cell proliferation [9]. It is approved for use in unresectable gastrointestinal stromal tumors (GIST), which have progressed on imatinib and sunitinib. It is also approved for metastatic colorectal cancer previously treated with fluoropyrimidine, oxaliplatin, and irinotecan-containing chemotherapy regimens, anti-vascular endothelial growth factor (VEGF) therapy and if KRAS wild-type, anti-epidermal growth factor receptor (EGFR) therapy [10]. Grothey et al. in a phase 3 trial for metastatic colorectal cancer compared regorafenib to placebo. Side effects included elevated bilirubin, AST and ALT levels in 2% vs 1% of patients, respectively. Severe drug-induced liver injury was seen only in the regorafenib group, and it can be life-threatening [11]. In a phase 3 trial by Demetri et al. for advanced GIST, regorafenib was compared to placebo. One death due to hepatic failure was described [12]. Regorafenib carries an FDA black box warning for hepatotoxicity. It is recommended the dose be reduced from 160 mg to 120 mg for grade 3 AST/ALT elevations. Regorafenib should be discontinued if AST/ALT are >20 times ULN, AST/ALT >3 times ULN and a bilirubin >2 times ULN, any reoccurrence of AST/ALT >5 times ULN with a bilirubin > 2 times ULN; reoccurrence of AST/ALT >5 times ULN on a dose of 120 mg, or a grade 4 adverse reaction [13].

Irinotecan

Irinotecan is a topoisomerase I inhibitor. It binds to the topoisomerase DNA cleavage complex and leads to single-strand breaks of DNA, resulting in cell death. It is approved as first-line therapy in combination with 5-fluorouracil and leucovorin in patients with metastatic carcinoma of the colon or rectum, and for patients with metastatic colon or rectal carcinoma that has recurred or progressed following 5-fluorouracil-based therapy [14]. The pharmacokinetics of irinotecan in hepatic impairment were studied by Raymon et al. Patients were divided into four groups: group 1 – bilirubin within normal range; group 2 – bilirubin 1 to 1.5 times ULN; group 3 – bilirubin 1.51 to 3 times ULN; group 4 – bilirubin >3.1 times ULN. In groups 1 and 2 the starting dose was 350 mg/m^2^. In groups 3 and 4, the starting doses were 175 and 100 mg/m^2^ respectively. The results showed that the recommended dose in hepatic impairment with a bilirubin level ≤ 1.5 times ULN is 350 mg/m^2^ and 1.51 to 3.0 times ULN is 200 mg/m^2^ [15]. The dosage recommendations for irinotecan are to reduce the initial dose by one dose level, from 125 to 100 mg/m^2^ weekly, and from 350 to 300 mg/m^2^ every three weeks when increased bilirubin levels are present. When irinotecan is combined with 5-fluorouracil and leucovorin with a bilirubin level <2 mg/dL, it is recommended to initiate treatment with a one level dose reduction [16].

Everolimus

Everolimus is an oral mammalian target of rapamycin (mTOR) inhibitor [17]. It is FDA approved for pancreatic neuroendocrine tumors that are unresectable, locally advanced, or metastatic [18]. It is extensively metabolized by the cytochrome P450 system and p-glycoprotein, and liver injury can occur due to everolimus, or its toxic intermediate [19]. In a phase 3 trial, everolimus was compared with placebo in patients with advanced neuroendocrine pancreatic tumors. Less than 1% of the patients had increased AST and ALT levels in the everolimus group [17]. Peveling-Oberhag et al. studied the pharmacokinetics of everolimus in patients with hepatic impairment. They concluded that the dose of everolimus should be decreased to 7.5 mg in mild and 5 mg in moderate hepatic impairment. For patients with severe hepatic impairment, everolimus is not recommended, but a dose of 2.5 mg can be administered if the benefit outweighs the risk [20].

Gemcitabine

Gemcitabine is approved as a first-line treatment for locally advanced (unresectable stage II or stage III) or metastatic (stage IV) adenocarcinoma of the pancreas [21]. It is a pyrimidine analogue related to cytarabine. It is metabolized to form the nucleotide gemcitabine diphosphate (dFdCDP) and triphosphate (dFdCTP), which are then incorporated into DNA, and block DNA synthesis resulting in cell death [22]. Venook et al. conducted a phase I pharmacokinetic trial of gemcitabine in patients with hepatic or renal dysfunction. They divided patients into three groups; group 1 – AST levels ≤2 times normal and bilirubin levels <1.6 mg/dL; group 2 – bilirubin level of 1.6 to 7 mg/dL; and group 3 – creatinine level of 1.6 to 5 mg/dL. They assessed 40 patients for toxicity. The results showed that there was a transient elevation of transaminases, which was not dose-limiting. However, patients with elevated bilirubin levels had further deterioration in liver function. They recommended a dose reduction of gemcitabine in patients with elevated bilirubin levels [23]. Likewise, a retrospective study at the Medical University of South Carolina reviewed the charts of seven patients with elevated bilirubin levels >4 mg/dL who were given gemcitabine at a dose of 1000 mg/m^2^. One patient developed thrombocytopenia requiring that gemcitabine be discontinued. They concluded that no dose reduction is required for patients with liver dysfunction, unless the bilirubin is elevated [24]. Based on these trials, patients who have a total bilirubin level >1.6 mg/dL, should have a starting dose of 800 mg/m^2^, and if tolerated, the dose can be escalated [22].

Paclitaxel Albumin-Stabilized Nanoparticle

Paclitaxel albumin-stabilized nanoparticle (nab-paclitaxel) is approved by the FDA for metastatic pancreatic cancer in combination with gemcitabine. It inhibits cellular mitosis by binding to microtubulin and disrupting the cytoskeleton of the cancer cells. Its mechanism of hepatic injury is also probably due to its effect on the microtubule function of hepatocytes [25]. A phase 3 clinical trial in 861 patients with advanced pancreatic cancer was performed. Patients were divided into a nab-paclitaxel and gemcitabine group (431) and a gemcitabine alone group (430), all had adequate hepatic function, and bilirubin levels at or below ULN. Serious hepatotoxicity was not reported [26]. Studies in patients with moderate to severe hepatic impairment have not been conducted.

Sunitinib Malate

Sunitinib malate is FDA approved for progressive well-differentiated pancreatic neuroendocrine tumors that are unresectable, locally advanced and metastatic [27]. It is a tyrosine kinase inhibitor that blocks the activity of VEGFR2, PDGFR b, and c-kit, thereby preventing angiogenesis and cellular proliferation [28]. It is metabolized by the CYP 3A4 pathway and can cause liver injury through a toxic metabolite [29]. It carries an FDA black box warning of hepatotoxicity. It is recommended liver function tests be drawn prior to starting treatment, during each cycle of treatment and as clinically indicated [30]. Bello et al. conducted an open-label study for patients with normal, mild or moderate hepatic impairment who were given sunitinib 50 mg daily. They looked at the pharmacokinetics of sunitinib and its metabolite and saw no difference in the systemic exposure between patients with normal liver function and those with mild or moderate hepatic impairment [31].

Erlotinib

Erlotinib is an EGFR tyrosine kinase inhibitor. It is FDA approved in combination with gemcitabine for locally advanced, unresectable, or metastatic pancreatic cancer [32]. It causes direct liver injury as it is metabolized by the cytochrome P450 system in the liver [33]. In a phase 3 trial, erlotinib plus gemcitabine was compared with gemcitabine alone in 569 patients with advanced pancreatic cancer. Grade 4 hepatotoxicity including elevated bilirubin, AST, ALT levels occurred in <1% of patients in the erlotinib and gemcitabine group [34]. It is recommended to discontinue treatment with erlotinib in patients with a serum bilirubin >2 times ULN, and/or serum transaminases >3 times ULN; or the bilirubin level is three times ULN or transaminases are five times ULN [35] (Table 2).

**Table 2:** Medications that require dose adjustments for hepatic impairment. FDA: Food and Drug Administration; ULN: Upper limit of normal; AST: Aspartate transaminase; ALT: Alanine transaminase; VEGFR: Vascular endothelin growth factor receptor; GIST: Gastrointestinal stromal tumor; PDGFR: Platelet-derived growth factor receptor; EGFR: Epidermal growth factor receptor.

Medication	Drug Class	FDA approved indications	Hepatic Impairment	Dosage Adjustment
5-fluorouracil	-Pyrimidine analogue	-Colorectal adenocarcinoma -Pancreatic adenocarcinoma	Bilirubin <2 x ULN AST/ALT 3-5 x ULN	Reduce dose to 75%
			Bilirubin 2-4 x ULN AST/ALT 5-10 x ULN	Reduce dose to 50-75%
			Bilirubin 3-4 x ULN AST/ALT	Discontinue
Capecitabine	-Prodrug of 5-fluorouracil	-Adjuvant colon cancer (Duke’s C) -First line treatment for metastatic colorectal cancer	Mild to moderate	None
			Severe	Discontinue until bilirubin <3 x ULN
Regorafenib	-Multi-kinase inhibitor -inhibits VEGFR 2 and 3, Ret, Kit, PDGFR and Raf kinases	-Unresectable GIST, which has progressed on imatinib and sunitinib -Metastatic colorectal cancer previously treated with fluoropyrimidine, oxaliplatin, irinotecan chemotherapy, anti-VEGF therapy and if KRAS wild-type, anti-EGFR therapy	Grade 3 AST/ALT elevation	Reduce dose to 120 mg
			-AST/ALT >20 x ULN -AST/ALT >3 x ULN with bilirubin >2 x ULN -AST/ALT >5 x ULN on 120 mg -Grade 4	Discontinue
Irinotecan	-Topoisomerase I inhibitor	-First-line with 5-fluorouracil and leucovorin in metastatic colorectal carcinoma -Metastatic colorectal carcinoma that has recurred or progressed following 5-fluorouracil therapy	Bilirubin >2 x ULN	Not studied in clinical trials
			Bilirubin ≤2 x ULN	Reduce initial dose by one dose level -from 125 to 100 mg/m^2 ^weekly -from 350 to 300 mg/m^2^ every three weeks
Everolimus	-mTOR inhibitor	-Pancreatic neuroendocrine tumors (unresectable, locally advanced, or metastatic)	Mild	Reduce to 7.5 mg
			Moderate	Reduce to 5 mg
			Severe	2.5 mg if benefit outweighs risk
Gemcitabine	-Pyrimidine analogue	-First line for locally advanced (unresectable stage II or stage III) or metastatic (stage IV) adenocarcinoma of the pancreas	Bilirubin >1.6 mg/dL	Starting dose of 800 mg/m^2^
Paclitaxel albumin-stabilized nanoparticle	-Mitotic inhibitor	-Metastatic pancreatic cancer in combination with gemcitabine	Normal to mild	Administer at 125 mg/m^2^
			Moderate to severe	Not studied in clinical trials
Sunitinib malate	-Tyrosine kinase inhibitor -inhibits VEGFR2, PDGFR b, c-kit	-Progressive well-differentiated pancreatic neuroendocrine tumors (unresectable, locally advanced, metastatic)	Mild to moderate	None
			Severe	Not studied in clinical trials
Erlotinib	-EGFR inhibitor	-Combination with gemcitabine for locally advanced, unresectable, or metastatic pancreatic cancer	Bilirubin > 2 x , AST/ALT > 3 x in a patient with baseline hepatic impairment Or Bilirubin > 3 x ULN, AST/ALT 5 x ULN	Hold/discontinue

## Conclusions

Dosage adjustment of medication is sometimes necessary in colorectal and pancreatic cancer patients with hepatic impairment in order to prevent serious adverse events. This review article discusses the chemotherapeutic agents and targeted therapies available for the treatment of colorectal and pancreatic cancer patients. It highlights the classes of these agents as well as the dosage adjustments required, when necessary, for hepatic impairment. Along with the dose adjustment at therapy initiation, it is recommended that hepatic function tests be done with each treatment cycle, and as clinically indicated. Further dose adjustments may be required in the setting of worsening liver function. For colorectal and pancreatic cancer patients with hepatic impairment, clinicians need to review the available therapeutic options, adjust their doses, in order to treat these patients effectively.
